# Consequences of exposure to prenatal famine on estimated glomerular filtration rate and risk of chronic kidney disease among survivors of the great Ethiopian famine (1983–85): a historical cohort study

**DOI:** 10.1186/s12937-021-00675-8

**Published:** 2021-03-02

**Authors:** Kalkidan Hassen Abate, Misra Abdulahi, Fedlu Abdulhay, Getachew Arage, Mohammed Mecha, Mohammed Yenuss, Habtamu Hassen, Tefera Belachew

**Affiliations:** 1 Department of Nutrition and Dietetics, Institute of Health, Jimma University, Jimma, Ethiopia; 2 Department of Population and Family Heath, Institute of Health, Jimma University, Jimma, Ethiopia; 3 Department of Obstetrics and Gynecology, Faculty of Medical Sciences, Jimma University, Jimma, Ethiopia; 4 Department of Nutrition and Dietetics, College of Health Sciences, DebreTabor University, Debre Tabor, Ethiopia; 5 Department of Internal Medicine, Faculty of Medical Sciences, Jimma University, Jimma, Ethiopia; 6 Department of Environmental Health Science, College of Health and Medical Sciences, Wollo University, Dessie, Ethiopia

**Keywords:** Prenatal famine, eGFR: chronic kidney disease

## Abstract

**Background:**

The impact of an adverse prenatal environment such as famine exposure on the development of adulthood non-communicable chronic illnesses, including diabetes and hypertension has been well articulated in the recent past and supported by evidence. However, there exist few longitudinal studies conducted on the long term consequences of prenatal famine exposure on adulthood kidney function. Hence, we set out to examine whether prenatal exposure to the Ethiopian Great Famine (1983–1985) was associated with changes in estimated glomerular filtration rate (eGFR) and the risk of developing chronic kidney disease (CKD) later in adult life.

**Methods:**

The study was conducted in 219 famine exposed and 222 non exposed cohorts in Raya Kobo district, North Wollo Zone, Northern Ethiopia. Estimated GFR was computed from standardized serum creatinine using the CKD Epidemiology Collaboration (CKD-EPI) equation. The definition of CKD includes those with an eGFR of less than 60 ml/min/1.73 m2 on at least in two occasions of 90 days apart (with or without markers of kidney damage). Linear and logistic regression analyses were employed to examine the independent effect of prenatal famine exposure on eGFR and CKD respectively.

**Results:**

The mean (SD) serum creatinine of exposed and non-exposed groups were 0.78 (0.2) and 0.75 (0.2) respectively. The mean (SD) eGFR of exposed groups was 107.95 (27.49) while the non-exposed 114.48 (24.81) ml/min. In linear regression, the unadjusted model to examine the association between famine exposure and eGFR resulted in a significant negative beta coefficient (β = − 0.124: 95% CI: − 11.43, − 1.64). Adjusting the exposure for outstanding covariates of kidney health, including systolic blood pressure, fasting blood sugar and blood glucose did not alter the inverse relationship (β = −.114 95% CI: − 10.84, − 1.17). In the unadjusted bivariate logistic regression model, famine exposure resulted in nearly 2.7 times higher odds of developing CKD (OR: 2.68, 95% CI: 1.16, 6.2). The odds remained equivalent after adjusting for systolic blood pressure, fasting blood glucose and body mass index (OR = 2.61: 95% CI: 1.120, 6.09).

**Conclusion:**

In the study setting, prenatal exposure to the Great Ethiopian Famine was associated with decreased eGFR and higher risk of developing CKD among survivors. These findings may imply that famine in early life may play a significant role in the development of kidney dysfunction in adulthood.

**Supplementary Information:**

The online version contains supplementary material available at 10.1186/s12937-021-00675-8.

## Background

*Mortality* and burden of *disease* related to suboptimal kidney functioning are the major contributors to global ill health [1]. The world health organization (WHO) reported an *estimated* 5–*10* million people die annually from *kidney disease* [2]. According to the Global Burden of Disease (GBD) estimates, in 2017 alone, with a global prevalence of 9.1%, chronic kidney disease (CKD) resulted in 1.2 million deaths [3]. Contrary to what the socio- demographic index (SDI) indicates, most of the burden of CKD disproportionately affects low-and middle-income countries, where detection rates remained low [1, 3, 4]. Despite one in three people in the general population are at increased risk of CKD, 90% of those with CKD are unaware of their condition [5].

Direct measurement of GFR to determine CKD status is complicated in clinical as well as survey settings as it requires substantial time and resource [3, 6, 7]. Alternatively, GFR can be estimated based on the plasma concentration of creatinine or cystatin C, while the latter is more reliable [8, 9]. Moreover, the estimated glomerular filtration rate (eGFR) is the best indicator to determine to the stages of kidney disease [7–9]. According to many guidelines, the definition of CKD includes those with an EGFR of less than 60 ml/min/1.73 m2 on at least two occasions of 90 days apart (with or without markers of kidney damage) [3–10].

Although often considered as a comorbidity of diabetes or hypertension, CKD has numerous complex causes [1–5]. Spanning in the life-course, important risk factors for kidney disease are documented as environmental, infections, and lifestyle factors [2–4]. Atypically, common etiologies in low-income countries have been documented as diarrheal diseases, HIV infection, low birth weight, malaria and preterm birth [2, 3]. Furthermore, data in recent decades also showed correlations between suboptimal kidney health among adults and their childhood adverse events such as prenatal famine exposure [11–18].

The impact of adverse prenatal famine or starvation on late adulthood chronic illness including diabetes, hypertension and CVD has been well articulated in the recent past and supported by evidence [14–16]. As it was best described by “The Barker hypothesis” (1990), an adverse nutrition in early life, including prenatally as measured by birth weight, increased susceptibility to the metabolic syndrome, which includes obesity, diabetes, insulin insensitivity, hypertension, and hyperlipidemia and complications that include coronary heart disease and stroke [16]. In line with Barker, the available famine studies also documented a higher proportion of diabetes, hypertension and changes in metabolic markers among survivors during adulthood [16, 19, 20]. However, only few longitudinal studies were conducted to investigate the long term consequence of perinatal famine on kidney function. Nonetheless, the available studies conducted among Chines and the Dutch famine survivors reported higher risk of CKD and or suboptimal kidney functional markers such as protein urea among prenatal famine exposed groups [11–14, 21].

If risk groups are identified early, chronic kidney disease can be prevented and or worsening of kidney function can be slowed or averted by known interventions [1–4]. The WHO indicates that the timely identification and management of population at risk of CKD represent the most effective strategy to address the growing global burden of chronic non-communicable diseases [2]. In line with the above, we used “the great Ethiopian famine” as a natural experiment setting to explore the impact of perinatal starvation on adulthood anthropometric metabolic and cognitive changes. In our earlier studies, in the same setting, we have reported the metabolic and cognitive changes associated with famine exposure during early childhood [22, 23]. Here, we aimed to assess whether prenatal exposure to the Ethiopian Great Famine (1983–1984) was associated with changes in glomerular filtration rate (GFR) and risk of CKD (EGFR< 60 ml/min per 1.73 m^2^) among adults in Wollo, North-East part of Ethiopia.

## Materials and methods

### Population and study design

The study was conducted in 36 Kebeles of Raya Kobo district, North Wollo Zone, Northern Ethiopia. The district covers an area of 2001.57 km^2^ and has a total population of 228,798 of which 147,837 are females [24]. A historical cohort study design was employed from March 15 to April 30, 2019, to investigate the effect of prenatal famine exposure on estimated glomerular filtration rate and risk of chronic kidney disease. Self-reported age and or birth date were used to categorize famine exposed and non exposed groups. Participants were categorized as prenatal exposed groups, if their age was 34 to 36 years old during the data collection period and or if their reported birth date was within 8th September 1983 to 30th August 1985. Non-exposed groups were participants aged 30 to 32 years and or their birth date was within 8th September 1987 to 8th October 1988 (suppl.1). To get the optimal washout of famine effect between the groups, participants who were born immediately after the end of the famine peak (between 8 September 1986 to 30 August 1987) were excluded. Further, exclusions were made for subjects with history of household displacement during the famine period, physical disability, including deformity (Kyphosis, Scoliosis, limb deformity) and pregnancy.

### Sample size and sampling procedures

The sample size was calculated by applying two population proportion assuming a prevalence of type two diabetes mellitus as proxy risk factor for CKD in fetal exposed group (22.6%) and non-exposed group (9.8%) [25]. Accordingly, the total sample size was 456 (228 exposed and 228 non- exposed). Multistage stratified random sampling technique was used to select the study participants. Details of participants’ selection procedure were depicted in supplementary figure (suppl.1).

### Study variables

The exposure variable was the great Ethiopian famine of the 1983–1985 G.C. Other sociodemographic, biochemical including fasting blood glucose, lipids and lipoproteins and anthropometric variables were also assessed. The outcome variable eGFR was computed from standardized serum creatinine using the CKD Epidemiology Collaboration (CKD-EPI) equation where eGFR (estimated glomerular filtration rate) expressed as ml/min/1.73 m^2^

$$ eGFR=141\times \min {\left( SCr/\kappa, 1\right)}^{\upalpha}\times \max {\left( SCr/\kappa, 1\right)}^{-1.209}\times {0.993}^{Age}\times 1.018\left[ iffemale\right]\times 1.159\left[ ifBlack\right] $$

Where S_Cr_ (standardized serum creatinine) in mg/dL, κ = 0.7 (females) or 0.9 (males), α = − 0.329 (females) or − 0.411 (males), min = indicates the minimum of S_Cr_/κ or 1, max = indicates the maximum of S_Cr_/κ or 1, age = years [8].

### Data collections and measurements

The data collection procedure and measurements were guided by standard operating procedures outlined by the WHO STEPwise approach to surveillance (STEPS) manual [26]. The data collection was carried out through an interview, biochemical test, blood pressure and anthropometric measurements.

#### Interview

Pretested structured questionnaire was used to collect sociodemographic data of the participants using face-to-face interview. The questionnaire was first prepared in English and then translated into Amharic (the local language) and back into English to ensure consistency. Eight trained clinical nurses collected the data.

#### Biochemical measurements

Fasting total cholesterol, plasma glucose, triglycerides and high-density lipoproteins were measured. Five milliliters of venous blood was collected in plane test tubes after overnight fasting participants (8–12 h). The analysis was made using A-25 bio-system® clinical chemistry analyzer. Low density lipoprotein (LDL) level was determined using Freidwald formula [27]. Standard operating procedures (SOP) were following to collect blood samples, perform laboratory analysis [26]. All laboratory analysis were conducted in Dessie branch of Amhara Public Health Institute (APHI) laboratory.

#### Anthropometric measurements

The height of participants was measured to the nearest 0.1 cm using a stadiometer (Seca®, Germany) with the subjects positioned at the Frankfurt Plane and the four points (heel, calf, buttocks and shoulder) touching the vertical stand and their shoes taken off. Weight was measured using portable battery operated Seca® digital scale. All anthropometric measurements were done in triplicate and the average value was used for further analyses [26]. The weigh scales were checked read zero and standardized using an object of known weight before measurement.

#### Blood pressure (BP)

Blood pressure was measured using a digital blood pressure apparatus in triplicate, with 5 minutes of rest in between measurements. The subsequent measurements were done 5 min apart. During data analysis, the mean of the second and third readings were calculated [26].

### Data processing and analysis

Data were double-entered using EpiData 3.1 and exported to SPSS for windows version 25 ([SPSS Inc. version 25, Chicago, Illinois] for cleaning and analysis. The data were cleaned by checking outliers and missing values. Categorical variables were described as frequencies and percentages and compared using the Pearson chi-square test. Continuous variables with a normal distribution were described using the relevant indicators of central tendency and spread (mean ± SD or median and IQR). Student’s t - test was used to evaluate the mean score difference between prenatal famine exposed and non-exposed cohorts.

Linear and logistic regressions were employed to examine the relationship between famine exposure in prenatal life and eGFR or CKD respectively. In order to account for the effect of outstanding biologic covariates four different regression models were evaluated. The first models in both regressions present unadjusted coefficients while model two, three and four were adjusted in a stepwise approach for fasting blood glucose, systolic hypertension and body mass index, respectively. Hosmer-Lemeshow test greater than 0.05 and maximum standard errors (SE) greater than 2 were used to check model fitness and multicollinearity, respectively. Potential effect modification was assessed by interaction terms. All analyses were two sided and *p* value of 0.05 was used to declare a significant difference. The results were presented as crude as well as adjusted odds ratio or beta coefficients and their 95%confidence intervals

### Ethics approval and consent to participate

Ethical clearance was obtained from Jimma University Institutional Review Board (IRB) as per protocol number IHRPGD/443/2018. Written informed consent was taken from each participant. The study participants were assured that they are free to withdraw their consent and discontinue participation without any form of prejudice. Privacy and confidentiality of collected data was ensured throughout the study. Copy of laboratory results were given to the respective participants and referral to the nearby public health facility were made for individuals with laboratory findings beyond the reference range.

## Results

**Fig. 1:**
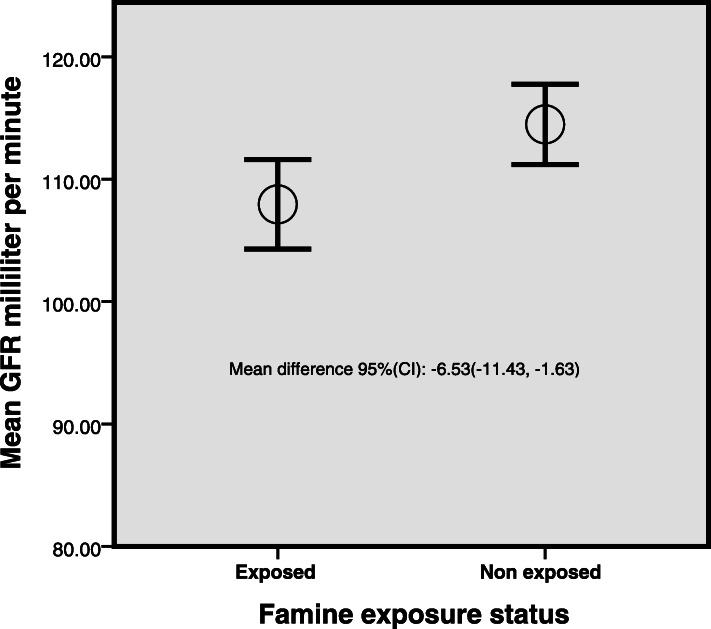
Comparison of GFR among prenatal famine exposed and non-exposed groups. Wollo, Ethiopia, Independent samples T test

The socio-demographic details of the study participant were published elsewhere [22] (sup. 1). Table 1 presents comparisons of the outstanding variables between 222 non exposed and 219 exposed individuals. The mean (SD) serum creatinine of exposed and non-exposed groups were 0.78 (0.2) and 0.75 (0.2) respectively. The mean (SD) eGFR of exposed groups was 107.95 (27.49) while the non-exposed 114.48 (24.81) (Fig. 1). There existed significant mean differences between the two groups in terms of eGFR, TG and Creatinine.

**Table 1:** Comparison of prenatal famine exposed and non-exposed groups on selected parameters. Wollo, Ethiopia, Independent samples T test

	Exposed Mean (SD)	Non exposed Mean (SD)	Mean difference 95(CI)
eGFR	107.95 (27.49)	114.48 (24.8)	−6.53(−11.43, − 1.63)*
BMI	23.03 (3.82)	22.64 (3.94)	0.39 (− 0.34, 1.11)
Triglycerides	107.26 (57)	91.67 (49.95)	17.33 (7.33, 27.33)*
Fasting blood glucose	86.44 (20.01)	82.91 (17.58)	3.13 (−0.39, 6.64)
Total cholesterol	138.17 (56.3)	130.69 (51.0)	7.48 (−2.57, 17.53)
HDL Cholesterol	44.21 ± 12.28	45.24 (12.31)	−0.83 (−3.09, 1.43)
Creatinine	0.78 (0.2)	0.75 (0.20)	0.03 (0.003, 0.07)*
Systolic BP (MmHg)	114.45 (12.50)	112.61 (11.99)	1.49 (−0.78, 3.75)

* Significant at *P* < 0.01, *eGFR* estimated glomerular filtration rate, *CI* confidence interval, *BMI* body mass index, *HDL* High density lipoprotein, *BP* blood pressure

Out of the famine exposed cohorts 20 participants and 8 for the non-exposed has CKD. Chi square test indicates a significant difference between the groups’ in terms of proportion of CKD (Table 2).

**Table 2:** Prevalence of CKD among prenatal famine exposed and non-exposed cohorts, Wollo, Ethiopia

			Non Exposed	Exposed	Total	*P*-value
CKD	Yes	Count	8	20	28	*0.029
		% within CKD	0.29	0.71	1.00	
	No	Count	214	199.00	413	
		% within CKD	0.52	0.48	1.00	
	Total	Count	222	219	441	

Chi square test: *significant at *P* < 0.05, *CKD* Chronic Kidney disease

In linear regression, unadjusted model to examine the association between famine exposure and eGFR resulted in significant negative beta coefficients (β = −.124: 95% CI: − 11.43, − 1.64) (model 1). In model 2, adjusted for fasting blood sugar, GFR is reduced by −.115 with the exposure (β = −.12, 95% CI:-10.88, − 1.17). In model three further adjustment was made for fasting blood glucose in addition for systolic blood pressure where exposure to famine remained to have a negative beta coefficient (β = −.109, 95% CI: − 10.62,-.89). The last model, adjusted for fasting blood sugar, systolic blood pressure and BMI resulted in a reduction of eGFR by −.114 with prenatal famine (β = −.114, 95% CI:-10.84, − 1.17).) (Table 3).

**Table 3:** Association of prenatal famine exposure and eGFR among adults, a linear regression analysis, Wollo, Ethiopia

Models	Βeta	95.0% CI
Model 1 (unadjusted)	−.124	−11.43, −1.63*
Model 2 (Adjusted for FBG)	−.115	−10.88, − 1.17**
Model 3 (Adjusted for FBG and systolic BP)	−.109	− 10.62, −.891**
Model 4 (Adjusted for FBG, systolic BP and BMI)	−.114	− 10.84,-1.17**

*Significant at *P* < 0.01, **significant at *P* < 0.05, fasting blood sugar, *BMI* body mass index, *BP* blood pressure

In binary logestic regression analysis, unadjusted model to examine the association between famine exposure and developing CKD resulted in positive odds, where exposure to famine resulted in nearly 2.7 times increased likelihood of having CKD (OR: 2.7, 95% CI: 1.16, 6.2) (model 1). Model two and three also showed in almost equivalent positive odds of having CKD with exposure (OR 2.63, 95% CI: 1.13, 6.13) and (OR: 2.61, 95% CI: 1.12, 6.11) respectively. In the final model, adjusted for systolic BP, fasting blood sugar and BMI resulted in equivalent odds with model 2 and 3; OR (CI): 2.61 95% CI: (1.12, 6.09) (Table 4).

**Table 4:** Association of prenatal famine exposure and CKD among adults, a linear regression analysis, Wollo, Ethiopia

Models	Beta	95.0% CI
Model 1 (unadjusted)	2.68	1.16, 6.24*
Model 2 (Adjusted for FBG)	2.63	1.13, 6.12*
Model 3 (Adjusted for FBG and systolic BP)	2.61	1.12, 6.10*
Model 4 (Adjusted for FBG, systolic BP and BMI)	2.61	1.12, 6.09*

*significant at *P* < 0.05, *FBG* fasting blood sugar, *BMI* body mass index, *BP* blood pressure

## Discussion

In our previous studies in the same study setting, we have reported the positive association between prenatal exposure to famine in early life and the risk of metabolic syndrome and cognitive malfunctioning late in adulthood [22, 23]. Furthermore, we have also identified anthropometric assaults associated with prenatal famine exposure, such as a reduction in adulthood height and an increase waist to height ratio [22]. In the present study, we are reporting results that show exposure to famine during the fetal period significantly associated with declined eGFR and raised prevalence of CKD. These findings may provide further evidence on the impact of the adverse intrauterine environment on adulthood kidney health.

The present finding indicates that exposure to prenatal famine had an association with the reduction in eGFR. We also found that, compared to non-exposed groups, adults who were exposed to famine in their fetal period had a higher proportion of CKD. In both of the above case scenarios, adjusting for classic outstanding covariates of CKD [2–4] (fasting blood glucose, BMI and systolic blood pressure) did not alter this association. This phenomenon may indicate the independence of the association between fetal malnutrition and impaired kidney function during adulthood.

Our findings were consistent with the Chines famine study, which reported lower EGFR (Beta = − 1.47, 95%CI − 2.81, − 1.13] and greater risk of having CKD (OR 2.85, 95%CI 1.25, 6.50) among famine exposed groups compared to controls [12]. However, the effect measures in the Chines famine study appear larger compared to our findings. This could be explained by the difference in the participant characteristics of the two studies where the Chines famine cohorts were older than our participants. Interestingly, surrogate to our eGFR marker, the Dutch famine study reported two fold of microalbuminuria among famine exposed cohorts during mid-gestation (OR 2.1, 95% CI 1.0, 4.3) [21]. A similar finding was also reported from another Chinese famine study which indicate a positive association of famine exposure with having higher protein urea (OR 1.54, 95% CI 1.04, 2.28) [14]. Corroborating the above findings, a recent systematic review reported unfavorable changes with kidney structure and function, measured by kidney volume, proteinuria, eGFR and mean creatinine clearance in the offspring of mothers with folate, vitamin A, and total energy deficiencies during pregnancy [28].

Apart from observational studies, findings from reviews of animal experimental studies reported that exposure to maternal global nutritional restriction during pregnancy to have unfavorable effects such as decreased kidney weight, lower nephron endowment, larger glomerular size, and lower GFR [29]. Taking low birth weight as a surrogate to nutrition restrictions in human, large scale studies such as the Helsinki Birth Cohort Study (1924–1944) reported a positive association of smaller body size at birth with increased risk for developing CKD [30]. Similarly, in many other studies, reductions in kidney mass, volume or nephron number were reported as adverse outcomes associated with low birth weight [31–34]. Nonetheless, as described in the recent systematic review of 34 studies, there remained reports of null effect of low birth weight on nephrogenesis, which may warrant the need for further investigation [35].

The implication of our findings should be interpreted considering the prevalent under nutrition in low income countries, which could have close or similar consequences on the health of the kidney during adulthood. Careful monitoring of CKD in hunger spots should be critical undertaking through early kidney disease detection programs and appropriate treatment of CKD risk factors such as obesity, high systolic blood pressure and elevated glucose levels.

This study has some limitations. Fasting blood glucose and creatinine was obtained based on a single measurement, so the prevalence of CKD might be overestimated. We also estimated GFR using CKD-EPI equation as we don’t have any locally adapted equation. Furthermore, due to paucity of data we have not included other important prenatal factors such as as gestational age, maternal nutritional and health conditions, mother’s smoking and drinking habits.

## Conclusion

Prenatal exposure to the Great Ethiopian Famine of 1984–85 was associated with decreased eGFR and greater risk of CKD among survivors. These findings may imply that famine in early life may play a significant role in the development of kidney dysfunction late in adulthood.

## Supplementary Information

**Additional file 1: Supp. Table 1.** Background characteristics of Ethiopian great famine exposed and non-exposed groups in North Wollo Zone, Raya Kobo district, Northeast Ethiopia, 2019. **Supp. Figure 1.** Flow diagram representing sample recruitment.

## Data Availability

The data supporting the conclusions of this article is included within the article (and its Additional file.

## Abbreviations

CI: Confidence interval
OR: Odds ratio
CKD: Chronic kidney disease
eGFR: estimated glomerular filtration rate
BMI: Body Mass Index
SD: Standard Deviation
FBG: Fasting blood sugar
SBP: Systolic BP
