# Supplementary material for: Consequences of exposure to prenatal famine on estimated glomerular filtration rate and risk of chronic kidney disease among survivors of the great Ethiopian famine (1983–85): a historical cohort study
Source: Nutr J. 2021 Mar 2;20:19. doi: 10.1186/s12937-021-00675-8 (PMC7927414; doi:10.1186/s12937-021-00675-8)
Supplement: Supplementary file 1 — Additional file 1: Supp. Table 1. Background characteristics of Ethiopian great famine exposed and non-exposed groups in North Wollo Zone, Raya Kobo district, Northeast Ethiopia, 2019. Supp. Figure 1. Flow diagram representing sample recruitment. [file 12937_2021_675_MOESM1_ESM.docx]

| **Supp. Table 1**. Background characteristics of Ethiopian great famine exposed and non-exposed groups in North Wollo Zone, Raya Kobo district, Northeast Ethiopia, 2019. | | | |
| --- | --- | --- | --- |
| Variables | Prenatal exposed  n = 222 | Non- exposed  n = 225 | *P-value* |
| Age, (years), mean ± SD | 35.14 ± 0.86 | 31.25 ± 0.65 | < 0.001^*^ |
| Sex, n (%) | | | |
| Female | 135 (60.8) | 123 (54.6) | 0.04^*^ |
| Male | 87 (39. 2) | 102 (45.5) |  |
| Residence, n (%) | | | |
| Urban | 42 (18. 2 ) | 42 (18.6) | 0.05^*^ |
| Rural | 180 (81.2) | 183 (81.3) |  |
| Educational status, n (%) | | | |
| Cannot read and write | 84 (37.8) | 53 (23.5) | 0.02^*^ |
| Primary school | 55 (24.7) | 42 (18.6) |  |
| Secondary school | 46 (20.7) | 67 (29.7) |  |
| Above secondary school | 37 (16.8) | 63 (28.0) |  |
| Household wealth index, n (%) | | | |
| Low | 74 (33.3) | 67 (29.7) | 0.64 |
| Medium | 110 (49.5) | 121 (53.7) |  |
| High | 38 (17.2.7) | 37(16.4) |  |
| Marital status, n (%) | | | |
| Single | 33 (14.8) | 59 (26. 2) | 0.04^*^ |
| Married | 145 (65.3) | 145 (64. 4) |  |
| Divorced/Widowed | 44 (19.5) | 21 (9.3) |  |
| Physical activity level, n (%) | | | 0.06 |
| Low | 4 (1.8%) | 8 (3.5%) |  |
| Moderate | 39 (17.5%) | 24 (10.6%) |  |
| High | 179 (80.6%) | 193 (85.7%) |  |
| Dietary pattern, n (%) | | | |
| Healthy | 63 (28.4%) | 76 (33.7%) | 0.22 |
| Unhealthy | 159 (71.6%) | 149 (66.2%) |  |
| Currently smoking, n (%) | | | |
| Yes | 40 (18.2%) | 31 (13.7%) | 0.20 |
| No | 182 (81.8%) | 194 (86.3%) |  |
| Currently drinking alcohol, n (%) | | | |
| Yes | 85 (46.7%) | 97 (18.2%) | 0.29 |
| No | 137 (51.7%) | 128 (18.2%) |  |
| P-value—represents Independent Samples t-tests for continuous variables or χ2-test for categorical variables, * Statistical significance | | | |

Registration was carried out to prepared sampling frame

Total subjects (n **456**)

Proportional allocation to selected kebeles then participants are selected by simple random sampling techniques

Exclusion

Missing values of laboratory data (n **9**)

Participants included in the study (n **447**)

1. Non- exposed groups (n **225**)
2. Prenatal-exposed groups (n **222**)

Supp. **Figure** **1**. Flow diagram representing sample recruitment.
